# Effect of Delays in Concordant Antibiotic Treatment on Mortality in Patients With Hospital-Acquired *Acinetobacter* Species Bacteremia: Emulating a Target Randomized Trial With a 13-Year Retrospective Cohort

**DOI:** 10.1093/aje/kwab158

**Published:** 2021-05-27

**Authors:** Cherry Lim, Yin Mo, Prapit Teparrukkul, Maliwan Hongsuwan, Nicholas P J Day, Direk Limmathurotsakul, Ben S Cooper

**Keywords:** *Acinetobacter* species, bacteremia, causal inference, empirical antibiotic treatment, patient mortality

## Abstract

Delays in treating bacteremias with antibiotics to which the causative organism is susceptible are expected to adversely affect patient outcomes. Quantifying the impact of such delays to concordant treatment is important for decision-making about interventions to reduce the delays and for quantifying the burden of disease due to antimicrobial resistance. There are, however, potentially important biases to be addressed, including immortal time bias. We aimed to estimate the impact of delays in appropriate antibiotic treatment of patients with *Acinetobacter* species hospital-acquired bacteremia in Thailand on 30-day mortality by emulating a target trial using retrospective cohort data from Sunpasitthiprasong Hospital in 2003–2015. For each day, we defined treatment as concordant if the isolated organism was susceptible to at least 1 antibiotic given. Among 1,203 patients with *Acinetobacter* species hospital-acquired bacteremia, 682 had 1 or more days of delays to concordant treatment. Surprisingly, crude 30-day mortality was lower in patients with delays of ≥3 days compared with those who had 1–2 days of delays. Accounting for confounders and immortal time bias resolved this paradox. Emulating a target trial, we found that these delays were associated with an absolute increase in expected 30-day mortality of 6.6% (95% confidence interval: 0.2, 13.0), from 33.8% to 40.4%.

## Abbreviations

AS-HAB: *Acinetobacter* species hospital-acquired bacteremia
AST: antimicrobial susceptibility testing
CCI: Charlson Comorbidity Index
CI: confidence interval
ICU: intensive care unit
IPW: inverse probability weight
MDR: multidrug resistant

Initial treatment for suspected bloodstream infections in hospital settings is usually given before the causative organism or its susceptibility to antibiotics is known. Such empirical antibiotic treatment is invariably designed to have broad coverage so there is a high chance that the causative organism is susceptible to one of the prescribed antibiotics; if it is susceptible, the treatment is said to be “concordant.” When designing empirical antibiotic regimes, considerations about concordance must be balanced against concerns about selection for resistance (1, 2). In settings where antimicrobial resistance is common, such empirical treatment is often “nonconcordant” (i.e., the causative organism is not susceptible to any of the antibiotics given). The resulting delay in the time until the patient receives appropriate antibiotics might lead to worse patient outcomes (3) and is a key mechanism by which antimicrobial resistance is thought to add to the burden of disease (i.e., health impact due to antibiotic-resistant infections) (2, 4).

In most high-income settings, a diagnostic microbiology laboratory will attempt to identify the causative organism and its susceptibility to different antibiotics. The process can take 2–4 days from the time the blood sample is taken to getting antibiotic susceptibility testing results, which might prompt a change in the antibiotics given to the patient (5). Empirical antibiotic treatment might also change prior to the availability or in the absence of antibiotic susceptibility results if the patient shows no clinical improvement (5). Many hospitals in low- and middle-income countries do not have access to a diagnostic microbiology service, and even when they do, the threshold of clinical suspicion for taking a blood culture is often higher than in high-income settings. Both of these factors can lead to longer delays to concordant antibiotic treatment. Quantifying the impact of delays in concordant antibiotic treatment on patient outcomes is therefore important for understanding the potential benefits of investment in microbiological diagnostic capacity, changes in blood culture practice, and improving empirical antibiotic prescribing policies. It is also important for understanding the potential benefit of new technologies that can reduce the time taken to obtain antibiotic susceptibility testing results.

Quantifying the causal effect of delays in concordant treatment on patient outcomes poses a number of challenges. A “gold standard” design would be to randomize patients to different delays until concordant treatment (6). However, it would clearly be unethical to intentionally delay the provision of appropriate antibiotic treatment. Therefore, analysis of observational data is likely to be the best alternative to address the question in practice. In addition, there are potential biases that can threaten the validity of inferences made from observational data, and consideration of many of these biases has been neglected in the considerable literature on the impact of delays in appropriate antibiotic treatment on patient outcomes. First, key baseline confounders need to be considered when studying the causal relationship between antibiotic treatment and patient in-hospital mortality, and should be adjusted for in the analysis. Second, antibiotic treatment can change over the course of an infection (time-varying exposure), and these changes might be related to the time-varying clinical severity of the patient, which could be a confounder for the current treatment and a mediator for the future treatment. Finally, observational studies are vulnerable to bias when patients are classified into exposure groups after time zero (7–9). As explained in detail in Hernán et al. (8) these biases can be avoided by specifying a target trial. The hypothetical target trial does not need to be feasible in practice given current technology, ethical concerns, or financial constraints. Rather, specifying a target trial should be considered as an exercise to make sure that the observational data is designed and analyzed in the most appropriate way possible (8, 10). Note that even when a randomized trial has been performed, analysis of observational data informed by a target trial will often be of considerable value, for example, due to the much larger sample sizes that can be achieved and the fact that the study population might be more representative of the population to which we wish to generalize (11).

The aims of this work are to demonstrate how an analysis that attempts to emulate a target randomized trial can address these problems, and to apply the target trial emulation methodology to quantify the impact of delays in concordant antibiotics for treating *Acinetobacter* species bloodstream infections in Northeast Thailand. *Acinetobacter* species are among the leading causes of bloodstream infections in Thailand, and multidrug-resistant (MDR) *Acinetobacter* species have been estimated to cause 15,000 excess death per year (12–14).

## METHODS

### Emulating 2 target trials

Trying to make causal inferences from observational data can be thought of as attempting to emulate a target randomized trial (6). A framework to make the target trial explicit to guide analytical approaches and to help avoid common methodologic pitfalls has been outlined by Hernán and Robins (6). Here, to apply the framework to the study data we first defined 2 hypothetical target trials to address related questions about the effect of delays to concordant antibiotic treatment on patient outcomes. Both would be unethical to perform in practice, but they are intended to guide the analysis (6). Summaries of these protocols are in Web Tables 1 and 2 (available at https://doi.org/10.1093/aje/kwab158). In brief, eligibility criteria are the same in both trials: patients of any age hospitalized for at least 2 calendar days when a blood sample was collected, with *Acinetobacter* species identified from the blood sample. The first target trial compares 2 treatment strategies: at least 1 day of delay in concordant antibiotic treatment versus concordant treatment on the same day as blood sample collection (i.e., no delay). In the second target trial, patients are randomly assigned to one of 4 treatment strategies: 1) no delays, 2) 1 day of delay, 3) 2 days of delay, and 4) at least 3 days of delay in receiving concordant antibiotic treatment. For both target trials, patients are randomly assigned to the treatment strategies on the date of blood sample collection, and the concordant antibiotic prescribed would be determined by physician preference (allowing physicians to select antibiotic regimen is a common approach in antibiotic treatment duration trials (15)). The follow-up period starts at randomization and ends at the day of discharge from the hospital, the day of death within the hospital, or 30 days after randomization, whichever occurs first. The primary outcome of both trials is survival status at the end of follow-up. Note that there are no practical reasons to prevent such a trial being performed now for an organism such as *Mycobacterium tuberculosis* where the antibiotic resistance phenotype can be reliably determined within a few hours of taking a sample (16), but the technology to reliably predict antibiotic susceptibility in *Acinetobacter* species from the genotype has not yet been developed. The causal contrasts of interest are the per-protocol effect. We focus on the concordance of empirical antibiotics prescribed within the first 3 days after a blood sample is taken for microbiology culture without taking into account the dosing and frequency.

### Retrospective cohort data to emulate the target trials

We identified, from a 13-year retrospective cohort, patients with *Acinetobacter* species hospital-acquired bacteremia (AS-HAB) in Sunpasitthiprasong Hospital, Thailand. This is a provincial hospital with 1,500 beds. The hospital has a microbiology laboratory that performs microbial culture for isolate identification and antimicrobial susceptibility testing (AST) on a daily basis. The number of blood cultures performed in 2003 was 11,584 and in 2015 was 56,719. Patients were eligible for inclusion in this study if they had stayed in the hospital longer than 2 calendar days when a blood sample with growth of *Acinetobacter* species was collected. During the study period, bacterial culture was performed using standard methodologies for bacterial identification and AST based on guidelines of the Bureau of Laboratory Quality and Standards, Ministry of Public Health, Thailand (17). AST was performed using the disk diffusion method based on Clinical and Laboratory Standards Institute guidelines (18). The first episode of *Acinetobacter* species bloodstream infection per eligible patient was included in the analysis. If more than 1 isolate of *Acinetobacter* species with different susceptibility profiles was identified on the same day, only the isolate resistant to the largest number of antibiotics tested was included in the analysis. Data on antibiotic prescription, *International Classification of Diseases* codes (*Tenth Revision*), and demographics were collected for analysis.

The study was approved by the institutional review board of Sunpasitthiprasong Hospital (Ref. 005/2560). Strengthening the Reporting of Observational Studies in Epidemiology (STROBE) recommendations were followed (Web Table 3).

### Covariate selection

Potential confounders were identified using a directed acyclic graph to represent the presumed causal relationships between antibiotic treatment and patient mortality (Web Figure 1) (19). The key potential baseline confounders identified were severity of underlying illness (20), antibiotic resistance pattern of the *Acinetobacter* species isolated from the blood sample, year in which the blood samples were collected, and specialty of the attending physician. We used the time between date of admission and date of blood sample collection, admission to intensive care unit (ICU) on the day of hospital admission, the number of days on antibiotic treatment prior to blood sample collection, and age-stratified Charlson Comorbidity Index (CCI) score as surrogates of severity of underlying illness. The CCI scores were calculated from the *International Classification of Diseases* codes given to each patient by the attending physicians (21). MDR *Acinetobacter* species were defined as previously described (22). Because data on the specialty of the attending physician is not routinely collected in the electronic record, we used the department in which the patient was treated on the day of blood collection as a proxy variable. A time-varying confounder that could affect changes in empirical antibiotic treatment after blood sample collection is severity of infection, which could be affected by the history of treatment and itself can influence the decision on future treatment. The prescription of a vasopressor and transfer to ICU during the infection within the analysis time period were used to represent severity of the infection and both coded as binary time-varying variables. Patient demographic information, age, and sex were also included as covariates.

### Exposure groups

We considered any antibiotics prescribed within 3 days of the date of collection of the first blood sample from which *Acinetobacter* species was isolated to represent empirical treatment. This is because microbiological identification and AST usually require at least 3 days in hospitals in low- and middle-income countries. During the first 3 days of blood sample collection, appropriate treatment cannot be guaranteed for each and every case and might be influenced by patient characteristics, physician experiences with antibiotic prescription, and local epidemiology of antimicrobial resistance. An antibiotic regimen was defined as concordant if susceptibility testing indicated that the isolated *Acinetobacter* species was susceptible to at least 1 of the antibiotics given. Otherwise, the regimen was defined as discordant. Concordance of the antibiotic treatment was determined for each eligible patient on the day of blood sample collection (*t* = 0), 1 calendar day after blood sample collection (*t* = 1), and 2 calendar days after blood sample collection (*t* = 2). The 4 exposures groups were: 1) patients with no delays in concordant antibiotic treatment (i.e., patients who had concordant treatment on the day of blood sample collection); 2) patients with 1-day delay in concordant treatment (i.e., patients who did not have concordant antibiotic treatment at the least on *t* = 1, which included those who died or were discharged at *t* = 1); 3) patients with 2 days of delay in concordant treatment (i.e., patients who did not have concordant antibiotic treatment at the least on *t* = 1 and *t* = 2, which included those who died or were discharged at *t* = 2); and 4) patients with at least 3 days of delay in concordant treatment.

### Outcomes

The primary outcome was in-hospital all-cause mortality within 30 days of the date of collecting the first blood sample from which *Acinetobacter* species was isolated. If a patient was discharged alive from the hospital before day 30 or remained in the hospital on day 30, then the patient was considered to have survived in the primary analysis.

It is a common practice in Thailand for moribund patients to be discharged and to die at home. This might cause misclassification of outcomes. To address the issue and to see the impact of assuming no discharged patient dies within 30 days on the estimated effect, we performed a sensitivity analysis (Web Table 4 and 5), and patients who were either discharged without improvement or who rejected treatment and were discharged were classified into the group assumed to have died within 30 days.

### Statistical analysis

The effects of delays in concordant empirical antibiotic treatment on 30-day mortality were estimated using marginal structural models (23). We performed 2 analyses. The first analysis was to evaluate the impact of 1 or more days of delay in concordant antibiotic treatment (i.e., emulating the first target trial). The second analysis was to evaluate the effect of 1-day, 2-day, and ≥3-day delays in concordant antibiotic treatment (i.e., emulating the second target trial). Stabilized inverse probability weights (IPWs) were calculated for the 2 analyses independently based on methods described elsewhere (9). We applied 2 sets of IPWs to a marginal structural model. First, a propensity score for each patient was calculated to represent the probability of being prescribed with a concordant antibiotic treatment on the day of blood sample collection, 1 day after, and 2 days after. The propensity scores were then used to calculate stabilized IPWs. Second, to emulate the second target trial with treatment regimen assigned on enrollment, we used the 3-step procedure described by Hernán (9). Briefly, 3 duplicates of each observation were created to represent a data set in which counterfactual treatments were included. For instance, for a patient who had no concordant antibiotic on *t* = 0 and *t* = 1, and then died or was discharged from the hospital on *t* = 2, 3 clones were created, giving 4 observations for the patient (1 observed and 3 counterfactual treatments with each copy assigned to a different treatment strategy). Then those clones that deviated from their assigned strategy were artificially censored. In the example above, the clones that were assigned to no delays and 1 day of delay in concordant antibiotic treatment were censored because they deviated from their assigned strategy. Last, to address the selection bias due to the censoring process, the uncensored copies were given a weight that is equal to the inverse of the probability of being uncensored (9). We then applied a marginal structural logistic regression model with the stabilized IPWs to estimate the marginal probability of 30-day mortality under each treatment regimen.

Statistical analyses were performed using STATA, version 15.1 (StataCorp LLC, College Station, Texas). Detailed descriptions of the statistical analysis and code are provided in Web Appendix 1, Web Figure 2, and Web Table 6. A simulation study was also performed and confirmed that the procedure could recover the expected 30-day mortality associated with delays in concordant antibiotic treatment (Web Appendix 2).

We assessed our study using the Risk of Bias in Nonrandomized Studies of Interventions tools, ROBINS-I. The detailed assessment results are in Web Table 7.

## RESULTS

### Patients

**Figure 1:**
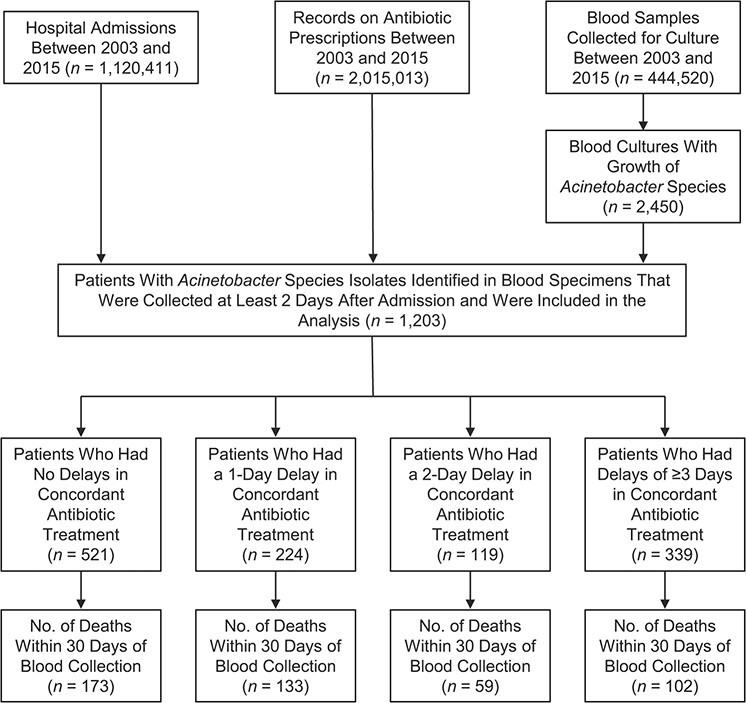
Flow chart of patients identified in the hospital microbiology database and included in an analysis to evaluate the impact of delays in concordant antibiotic treatment, Thailand, 2003–2015.

Between January 1, 2003, and December 31, 2015, 1,203 inpatients had a blood sample collected 2 or more days after hospital admission yielding *Acinetobacter* species (Figure 1). Among the eligible patient cohort, 521 patients had no delays in concordant antibiotic treatment (i.e., patients had concordant treatment on the day of blood sample collection); 224 patients had a 1-day delay; 119 patients had a 2-day delay; and 339 patients had 3 or more days of delay in concordant antibiotic treatment.

Patient characteristics varied across the 4 groups of exposures (Table 1). The proportion of patients admitted to ICU wards on the day of hospitalization was highest among those having a 1-day delay in concordant antibiotic treatment (159 of 224 patients; 71.0%), followed by those having a 2-day delay (72 of 119 patients, 60.5%). The proportion of patients with MDR *Acinetobacter* species isolates was highest among those who had a 1-day delay in concordant antibiotic treatment (206 of 224; 92.0%), followed by those who had 3 or more days of delay (302 of 339 patients; 89.1%). The majority of the MDR *Acinetobacter* species isolates were also resistant to carbapenem (877 of 975; 90.0%), and most of the non-MDR *Acinetobacter* species were susceptible to carbapenem (209 of 228; 91.7%).

**Table 1:** Characteristics of Patients With Hospital-Acquired *Acinetobacter* Species Bloodstream Infection That Were Included in the Analysis to Evaluate the Impact of Delays in Concordant Antibiotic Treatment, Thailand, 2003–2015

	**Delay in Concordant Antibiotic Treatment, days**							
**Covariate**	**None (*n* = 521)**		**1 (*n* = 224)**		**2 (*n* = 119)**		**3 or More (*n* = 339)**	
	**No.**	**%**	**No.**	**%**	**No.**	**%**	**No.**	**%**
Age, years^a^	54 (26–69)		57 (12–70)		51 (14–69)		54 (6–70)	
Female sex	212	40.7	103	46.0	56	47.1	143	42.2
Fluoroquinolone resistance	309	59.3	191	85.3	93	78.2	266	78.5
Carbapenem resistance	312	59.9	195	87.1	103	86.6	286	84.4
Multidrug resistance^b^	364	69.9	206	92.0	103	86.6	302	89.1
Age-adjusted CCI score on admission^a^	2 (0–4)		2 (0–5)		2 (0–4)		2 (0–4)	
Patients with vasopressor prescription on the day blood sample was collected for culture	211	40.5	129	57.6	50	42.0	103	30.4
Patients with vasopressor prescription on the second day after blood sample collected for culture	176	38.7^c^	22	40.7^d^	43	36.1	117	34.5
Patients with vasopressor prescription on third day after blood sample collection for culture	133	34.1^e^	17	34.7^f^	15	38.5^g^	97	28.6
Patients admitted to ICU on the day of hospitalization	285	54.7	159	71.0	72	60.5	162	47.8
Patients in the ICU on the day of blood sample collected for culture	304	58.4	156	69.6	70	58.8	191	56.3
Overall in-hospital mortality	194	37.2	137	61.2	59	49.6	120	35.4
30-day in-hospital mortality since blood collection	173	33.2	133	59.4	59	49.6	102	30.1
Length of hospital stay from admission to blood sample collection for culture, days^a^	9 (6–19)		8 (6–15)		9 (6–15)		10 (6–17)	
No. of days on antibiotic prior blood sample collection^a^	8 (5–15)		5 (0–10)		7 (3–12)		7 (4–14)	

Abbreviations: CCI, Charlson Comorbidity Index; ICU, intensive care unit.

^a^ Values are expressed as median (interquartile range). CCI scores were defined using codes from the *International Classification of Diseases, Tenth Revision* (21).

^b^ Multidrug resistance was defined as not being susceptible to ≥1 agent in ≥3 antimicrobial categories (22).

^c^ Denominator is 455; 66 patients with no delays in concordant antibiotic treatment were discharged from the hospital or died after *t* = 0.

^d^ Denominator is 54; 170 patients with a 1-day delay in concordant antibiotic treatment were discharged from the hospital or died after *t* = 0.

^e^ Denominator is 390; an additional 65 patients with no delays in concordant antibiotic treatment were discharged from the hospital or died after *t* = 1.

^f^ Denominator is 49; an additional 5 patients with a 1-day delay in concordant antibiotic treatment were discharged from the hospital or died after *t* = 1.

^g^ Denominator is 39; 80 patients with a 2-day delay in concordant antibiotic treatment were discharged from the hospital or died after *t* = 1.

The most commonly prescribed antibiotics on the day of blood sample collection were carbapenems (*n* = 312) followed by ceftazidime (*n* = 121). Of 467 patients who died within 30 days after blood sample collection, 294 patients did not have a concordant antibiotic prescription on the day of blood sample collection. Of those patients, 33.7% (99/294) had a prescription for a carbapenem, 16.0% (47/294) did not have an antibiotic prescription, and 9.9% (29/294) had a prescription for a third-generation cephalosporin. Of 736 patients who survived for at least 30 days after the first positive blood sample was collected, 24.2% (178/736) had a prescription for a carbapenem on the day of blood sample collection, 17.1% (126/736) had a prescription for a third-generation cephalosporin, and 10.1% (74/736) did not have an antibiotic prescription.

### Antibiotic treatment concordance on the day of blood sample collection and 30-day in-hospital mortality

Based on the analysis to emulate the first target trial, results showed that receiving concordant antibiotic treatment on the day of blood collection was associated with reduced 30-day mortality, after adjusting for the prespecified confounders. Patients given concordant antibiotic treatment on the day of blood collection had an expected 30-day mortality of 33.8% (95% confidence interval (CI): 29.1, 38.5), compared with an expected 30-day mortality of 40.4% (95% CI: 36.1, 44.7) in those not treated with concordant antibiotics. The absolute difference was 6.6% (95% CI: 0.2, 13.0).

### Days of delay in concordant antibiotic treatment and 30-day in-hospital mortality

**Figure 2:**
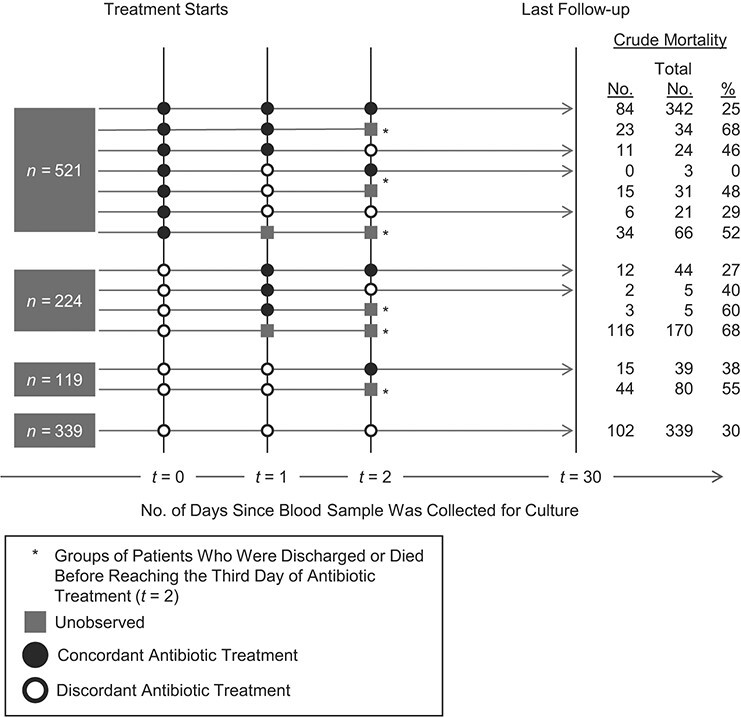
Regimen assignment, all-cause 30-day in-hospital mortality, and discharge patterns over 3 days after blood sample collection among the study cohort used for an analysis to evaluate the impact of delays in concordant antibiotic treatment, Thailand, 2003–2015. “Treatment starts” indicates the time at which an empirical antibiotic treatment would be initiated for a patient in a hypothetical randomized controlled trial. This is the time at which patients are enrolled into the study and randomized to one of the 4 treatment strategies.

In the analysis to emulate the second target trial, the crude 30-day in-hospital all-cause mortality was highest among those with a 1-day delay in concordant antibiotic treatment (133 of 224 patients; 59.4%), and lowest among those with 3 or more days of delays in concordant antibiotic treatment (102 of 339 patients; 30.1%) (Table 2). The discharge pattern of patients under different treatment groups varied over the 3 days of the initial treatment period (Figure 2). Among the 1,203 eligible patients, 236 (19.6%) either died or were discharged from the hospital 1 day after blood was collected for culture and, of those patients, 63.6% (150 out of 236) died within the hospital (Figure 2).

**Table 2:** Estimated Probability of 30-Day Mortality for Each Exposure Group of Patients Who Were Included in the Analysis to Evaluate the Impact of Delays in Concordant Antibiotic Treatment, Thailand, 2003–2015

**Delay toConcordant Antibiotic Treatment, days**	**Total No.**	**Crude 30-Day In-Hospital All-Cause Mortality**		**Expected 30-Day Mortality** ^**a**^	
		**No.**	**%**	**Point estimate, %**	**95% CI**
None	521	173	33.2	39.8	32.3, 47.2
1	224	133	59.4	42.8	29.8, 55.7
2	119	59	49.6	51.0	38.9, 63.1
≥3	339	102	30.1	40.9	36.0, 45.8

Abbreviation: CI, confidence interval

^a^ Estimated from a marginal structural model with stabilized inverse probability weights.

The marginal structural model adjusting for baseline confounders, time-varying confounders, and immortal time bias resolved paradoxical observations in the crude data (Table 2). While the crude analysis suggested that patients with 3 or more days of delays in concordant antibiotic treatment had the lowest mortality, the adjusted analysis found that the expected mortality was lowest if the patients had no delays, although we found no evidence of increasing mortality with increasing delays. However, uncertainty was large. Absolute differences in mortality between no delays in concordant antibiotic treatment and a 1-day delay, a 2-day delay, and 3 or more days of delay were 3.0% (95% CI: −12.0, 18.0); 11.3% (−3.0, 25.6); and 1.1% (−7.8, 10.0), respectively.

### Sensitivity analysis

Under the assumption that patients discharged within 30 days of the first positive blood culture either without improvement or having rejected treatment died within 30 days, a similar effect of delayed concordant treatment was observed. If patients were given a concordant antibiotic treatment on the day of blood sample collection, the expected marginal probability of developing a detrimental outcome (death or discharge without improvement) would be 58.9% (95% CI: 53.8, 63.9), which is lower than if they were not given a concordant antibiotic treatment (62.0%, 95% CI: 57.7, 66.4). The difference was 3.2% (95% CI: −3.5, 9.9). The estimated marginal probabilities of developing a detrimental outcome (death or discharge without improvement) within 30 days of blood collection were 64.6% (95% CI: 56.8, 72.4), 63.2% (95% CI: 50.4, 76.0), 68.4% (95% CI: 56.1, 80.7), 63.4% (95% CI: 58.5, 68.3) for no delays, a 1-day delay, a 2-day delay, and 3 or more days of delay in concordant antibiotic treatment, respectively (Web Table 4). The estimated impacts of the treatment regimens on detrimental outcomes were similar to the effects on 30-day in-hospital mortality (Web Table 5).

## DISCUSSION

After adjusting for measured confounders, we found that delays in concordant antibiotic treatment of 1 or more days were associated with an absolute increase of 6.6% in 30-day mortality from 33.8% to 40.4% in the first analysis attempting to emulate a target trial with 2 treatment arms. There was no evidence of a dose-response relationship between days of delays in concordant antibiotics and 30-day mortality in the second analysis.

AS-HAB is associated with increased patient mortality, especially in developing countries (12). The proportion of hospital-acquired *Acinetobacter* species bacteremias that are MDR can be as high as 75%, and attributable mortality has been estimated to range from 18% to 41% in developing countries (12–14, 24–27). Therapeutic options for treating MDR *Acinetobacter* species infections are limited. Carbapenem, colistin, and tigecycline are currently the last-resort antibiotics for drug-resistant *Acinetobacter* species bloodstream infection, and increasing resistance to these antibiotics has been reported in developing countries (28, 29). The spread of resistant pathogens can be fueled by the overuse of broad-spectrum antibiotics in hospital settings (2), and *Acinetobacter* species have an ability to assemble different mechanisms of resistance (12). Hence, time to initiation of antibiotic treatment in patients suspected of having bacterial infection is important both to control the spread of resistant infection and to save lives. Hospital antibiotic policies that minimize morbidity and mortality due to infections, while preserving the effectiveness of antimicrobial agents for treatment purposes, are important in preventing the spread of microbial infections (1). Moreover, statistics on the impact of delays to concordant antibiotic treatment are important when estimating the potential benefits of diagnostic stewardship and of the efforts to reduce the time taken to obtain AST results. Our estimates of the impact of delays in concordant empirical antibiotic treatment on patient mortality among those with hospital-acquired *Acinetobacter* species bloodstream infection will be of particular relevance in settings with a high incidence of drug-resistant *Acinetobacter* species infection.

Immortal time bias is a common phenomenon and needs to be considered in studies comparing treatment regimens where the observed treatment durations vary (7, 8). In this study, empirical antibiotic treatment over a period of 3 days after blood collection was considered, and only patients who survived up to 3 days after blood collection could be classified into the group of “≥3 days of delays in concordant treatment.” Hence, by definition, they cannot have died within the first 3 days and for this time period they are effectively “immortal.” This bias will tend to make them appear to survive longer compared with the reference group (no delays in concordant antibiotic treatment). This is reflected in the paradoxical observation that the crude all-cause 30-day mortality was lowest among patients with ≥3 days of delay in concordant antibiotic treatment. This bias can be avoided (and the paradox resolved) by adopting the target trial emulation methodology as described by Hernán et al. (8) and employed in our analysis.

Previous analyses have evaluated the impact of delayed antibiotic treatment on outcomes for patients with bacteremia related to *Acinetobacter* species, but appropriate adjustment for biases has been lacking (30–37). A study in Taiwan on 252 patients with ICU-acquired bloodstream infections caused by *Acinetobacter baumannii* suggested appropriate antibiotic therapy (defined as administration of at least 1 antibiotic treatment that is appropriate in type, route, and dosage within 48 hours of bacteremia onset) reduced 14-day mortality (adjusted odds ratio was 0.22 (95% CI: 0.10, 0.50)) (34). The analysis used a multivariable logistic regression model adjusted for APACHE II score measured 2 days prior to bacteremia onset and for malignancy. In this study, among those with APACHE II scores of >35, more than 70% of patients died within 24 hours in the inappropriate antibiotic group, and in the appropriate treatment group no patient died within the initial 48-hour treatment period (34). Some of the reported differences in survival probability are therefore expected to be due to immortal time bias.

In most previous studies, antibiotic use has been considered as a binary variable and switching of antibiotic regimens due to changes in clinical symptoms has been neglected (5). In hospitals in resource-limited settings, antibiotics are sometimes prescribed even before a clinical specimen is taken for culture, and switching regimens in response to severity of infection is common (5). This change in regimen determined by clinical signs, if not adjusted for using appropriate methods for time-varying confounders, could also lead to biases (6, 23). Marginal structural models have been used to adjust for time-varying confounders in a previous study of the association between appropriate antibiotic treatment for bacteremia on the day the blood culture was taken and mortality and discharge (38). The previous study found no evidence for a protective effect of appropriate empirical antibiotic treatment on mortality and discharge, but confidence intervals were wide (38). Differences in bacterial species considered, patient characteristics and clinical setting make direct comparison with the current study inappropriate.

Our study has limitations. First, data on the severity of infections were not routinely collected, and this is typically the case in low- and middle-income country settings. We used admission to ICU and prescription of vasopressors as proxy variables for the severity of infection. These proxy variables will only imperfectly represent the severity of infection, and residual confounding is to be expected; however, both are specific in representing severe clinical conditions. Second, despite the relatively large sample size, the power to detect a dose-response relationship in the 4 regimens under evaluation might be low. This is reflected in the wide confidence intervals in our expected mortality under each regimen. Third, our emulation included only AS-HAB. Initiating empirical antibiotic treatment for patients with hospital-acquired sepsis would include both AS-HAB and non–AS-HAB. Nonetheless, point-of-care rapid diagnostic tests for AS-HAB with AST results are critically needed to differentiate from non–AS-HAB to avoid overuse of antibiotics that target non-MDR and MDR AS-HAB. Fourth, residual confounding factors could be present.

In conclusion, we observed a 6.6% (95% CI: 0.2, 13.0) absolute increase in mortality among patients with hospital-acquired *Acinetobacter* species bacteremia when concordant antibiotic treatment was delayed for 1 or more days. Accounting for confounding and immortal time biases is necessary when attempting to estimate causal effects of delayed concordant treatment and, in this case, helped resolve paradoxical results in the initial crude data analysis.

## Supplementary Material

Web_Material_kwab158
