# Supplementary material for: Effect of Delays in Concordant Antibiotic Treatment on Mortality in Patients With Hospital-Acquired Acinetobacter Species Bacteremia: Emulating a Target Randomized Trial With a 13-Year Retrospective Cohort
Source: Am J Epidemiol. 2021 May 27;190(11):2395–404. doi: 10.1093/aje/kwab158 (PMC8561124; doi:10.1093/aje/kwab158)
Supplement: Web_Material_kwab158 [file web_material_kwab158.pdf]

Web Material

Effect of Delays in Concordant Antibiotic Treatment on Mortality in Patients With Hospital-Acquired *Acinetobacter* Species Bacteremia: Emulating a Target Randomized Trial With a 13-Year Retrospective Cohort

Cherry Lim, Yin Mo, Prapit Teparrukkul, Maliwan Hongsuwan, Nicholas P. J. Day, Direk Limmathurotsakul, and Ben S. Cooper

Author affiliations: Mahidol Oxford Tropical Medicine Research Unit, Faculty of Tropical Medicine, Mahidol University, Thailand (Cherry Lim, Yin Mo, Maliwan Hongsuwan, Nicholas P.J. Day, Direk Limmathurotsakul, and Ben S Cooper); Centre for Tropical Medicine and Global Health, Nuffield Department of Medicine, University of Oxford, United Kingdom (Cherry Lim, Yin Mo, Nicholas P.J. Day, Direk Limmathurotsakul, and Ben S Cooper); Division of infectious disease, University Medicine Cluster, National University Hospital, Singapore, Singapore (Yin Mo); and Department of Internal Medicine, Sunpasitthiprasong Hospital, Ubon Ratchathani, Thailand (Prapit Teparrukkul)

Table of Contents

Web Table 1. A summary of the two Protocols of Target Trials to estimate the impact of delays in concordant antibiotic treatment on 30-day in-hospital mortality. This target trial is for comparing two treatment strategies. **3**

Web Table 2. A summary of the two Protocols of Target Trials to estimate the impact of delays in concordant antibiotic treatment on 30-day in-hospital mortality. This second target trial is for comparing four treatment strategies. **4**

Web Table 3. STROBE checklist. **5**

Web Figure 1. Directed acyclic graph (DAG) to represent the causal relationship between antibiotic treatment and patient mortality **7**

Web Table 4. The estimated probability of observing detrimental outcome within 30 days post blood collection under each exposure group. **8**

Web Table 5. The estimated effect of the four treatment regimens on 30-day in-hospital all-cause mortality and detrimental outcomes. **9**

Web Appendix 1. Statistical analysis and Stata code used to calculate the stabilized inverse probability weights for the marginal structural model. **10**

Web Figure 2. Diagnosis of the stabilized IPWs for assessing the exchangeability and positivity assumptions of the causal framework. **14**

Web Table 6. Patient characteristics of the four treatment regimens in the weighted sample. **15**

Web Appendix 2. A simulation study to confirm that the three-step procedure could alleviate immortal time bias. **16**

Web Table 7. Assessing our current study and the four articles identified with a similar study objective using the Risk of Bias in Non-randomised Studies of Interventions (ROBINS-I). . **19**

References **20**

Web Table 1. A summary of the two Protocols of Target Trials to estimate the impact of delays in concordant antibiotic treatment on 30-day in-hospital mortality. This target trial is for comparing two treatment strategies.

<u>Target trial 1</u>

**Protocol component**	**Description**
Eligibility criteria	Patients who had been hospitalized for at least 2 calendar days on the date of collection of a blood sample from which *Acinetobacter* spp. was identified.
Treatment strategies	Days of delay in receiving concordant antibiotic treatment. The two treatment strategies were i) initiate an antibiotic regimen that is concordant to the *Acinetobacter* spp. isolated from blood sample without delay (i.e. on the same day as the blood sample is taken); ii) initiate a treatment that does not cover the *Acinetobacter* spp. isolated from blood sample at baseline.
Assignment procedures	Patients will be randomly assigned to one of two the antibiotic treatment strategies at baseline (on the day of blood sample collection).
Follow-up period	Starts at randomization and ends at day of discharge from the hospital, day of death within the hospital, or 30 days post randomization, whichever occurs first.
Outcome	Survival status within 30 days post randomization.
Causal contrasts of interest	Per-protocol effect.
Analysis plan	Per-protocol effect estimate requires adjustments for pre-defined confounders and immortal-time bias using marginal structural models with inverse-probability weights.

Web Table 2. A summary of the two Protocols of Target Trials to estimate the impact of delays in concordant antibiotic treatment on 30-day in-hospital mortality. This second target trial is for comparing four treatment strategies.

**Protocol component**	**Description**
Eligibility criteria	Patients who had been hospitalized for at least 2 calendar days when a blood sample was collected and *Acinetobacter* spp. was identified.
Treatment strategies	Days of delay in receiving concordant antibiotic treatment. The four treatment strategies are: i) initiate an antibiotic treatment that is concordant to the *Acinetobacter* spp. isolated from blood sample without delay (i.e. on the same day as the blood sample is taken); ii) initiate a concordant antibiotic treatment one calendar day after the blood sample was taken; iii) initiate a concordant antibiotic treatment two calendar days after the blood sample was taken; and iv) no concordant antibiotic treatment for at least 3 days after blood sample collected. Patients are to keep the treatment strategies for at least 3 days post blood sample collection unless severity of infection increases during the treatment.
Assignment procedures	Patients will be randomly assigned to one of the four antibiotic treatment strategies at baseline (on the day of blood sample collection).
Follow-up period	Starts at randomization and ends at day of discharge from the hospital, day of death within the hospital, or 30 days post randomization, whichever occurs first.
Outcome	Survival status within 30 days post randomization.
Causal contrasts of interest	Per-protocol effect.
Analysis plan	Per-protocol effect estimate requires adjustments for pre-defined confounders and immortal-time bias using marginal structural models with inverse-probability weights.

Web Table 3. STROBE checklist.

	Item No	Recommendation	Page number
**Title and abstract**	1	(*a*) Indicate the study's design with a commonly used term in the title or the abstract	1
		(*b*) Provide in the abstract an informative and balanced summary of what was done and what was found	4
**Introduction**			
Background/rationale	2	Explain the scientific background and rationale for the investigation being reported	5-7
Objectives	3	State specific objectives, including any prespecified hypotheses	7
**Methods**			
Study design	4	Present key elements of study design early in the paper	8-9
Setting	5	Describe the setting, locations, and relevant dates, including periods of recruitment, exposure, follow-up, and data collection	9-10
Participants	6	(*a*) Give the eligibility criteria, and the sources and methods of selection of participants. Describe methods of follow-up	9-12
		(*b*) For matched studies, give matching criteria and number of exposed and unexposed	NA
Variables	7	Clearly define all outcomes, exposures, predictors, potential confounders, and effect modifiers. Give diagnostic criteria, if applicable	10-12
Data sources/ measurement	8*	For each variable of interest, give sources of data and details of methods of assessment (measurement). Describe comparability of assessment methods if there is more than one group	10
Bias	9	Describe any efforts to address potential sources of bias	12-13
Study size	10	Explain how the study size was arrived at	NA
Quantitative variables	11	Explain how quantitative variables were handled in the analyses. If applicable, describe which groupings were chosen and why	10-11
Statistical methods	12	(*a*) Describe all statistical methods, including those used to control for confounding	12-13
		(*b*) Describe any methods used to examine subgroups and interactions	NA
		(*c*) Explain how missing data were addressed	NA
		(*d*) If applicable, explain how loss to follow-up was addressed	NA
		(*e*) Describe any sensitivity analyses	12-13
**Results**			

Participants	13*	(a) Report numbers of individuals at each stage of study—eg numbers potentially eligible, examined for eligibility, confirmed eligible, included in the study, completing follow-up, and analysed	14
		(b) Give reasons for non-participation at each stage	Figure 1
		(c) Consider use of a flow diagram	Figure 1
Descriptive data	14*	(a) Give characteristics of study participants (eg demographic, clinical, social) and information on exposures and potential confounders	14
		(b) Indicate number of participants with missing data for each variable of interest	NA
		(c) Summarise follow-up time (eg, average and total amount)	Table 1
Outcome data	15*	Report numbers of outcome events or summary measures over time	14
Main results	16	(*a*) Give unadjusted estimates and, if applicable, confounder-adjusted estimates and their precision (eg, 95% confidence interval). Make clear which confounders were adjusted for and why they were included	14-16
		(*b*) Report category boundaries when continuous variables were categorized	Table 1
		(*c*) If relevant, consider translating estimates of relative risk into absolute risk for a meaningful time period	NA
Other analyses	17	Report other analyses done—eg analyses of subgroups and interactions, and sensitivity analyses	16
**Discussion**			
Key results	18	Summarise key results with reference to study objectives	17
Limitations	19	Discuss limitations of the study, taking into account sources of potential bias or imprecision. Discuss both direction and magnitude of any potential bias	17-19
Interpretation	20	Give a cautious overall interpretation of results considering objectives, limitations, multiplicity of analyses, results from similar studies, and other relevant evidence	17-19
Generalisability	21	Discuss the generalisability (external validity) of the study results	17-20
**Other information**			
Funding	22	Give the source of funding and the role of the funders for the present study and, if applicable, for the original study on which the present article is based	1

Web Figure 1. Directed acyclic graph (DAG) to represent the causal relationship between antibiotic treatment and patient mortality.

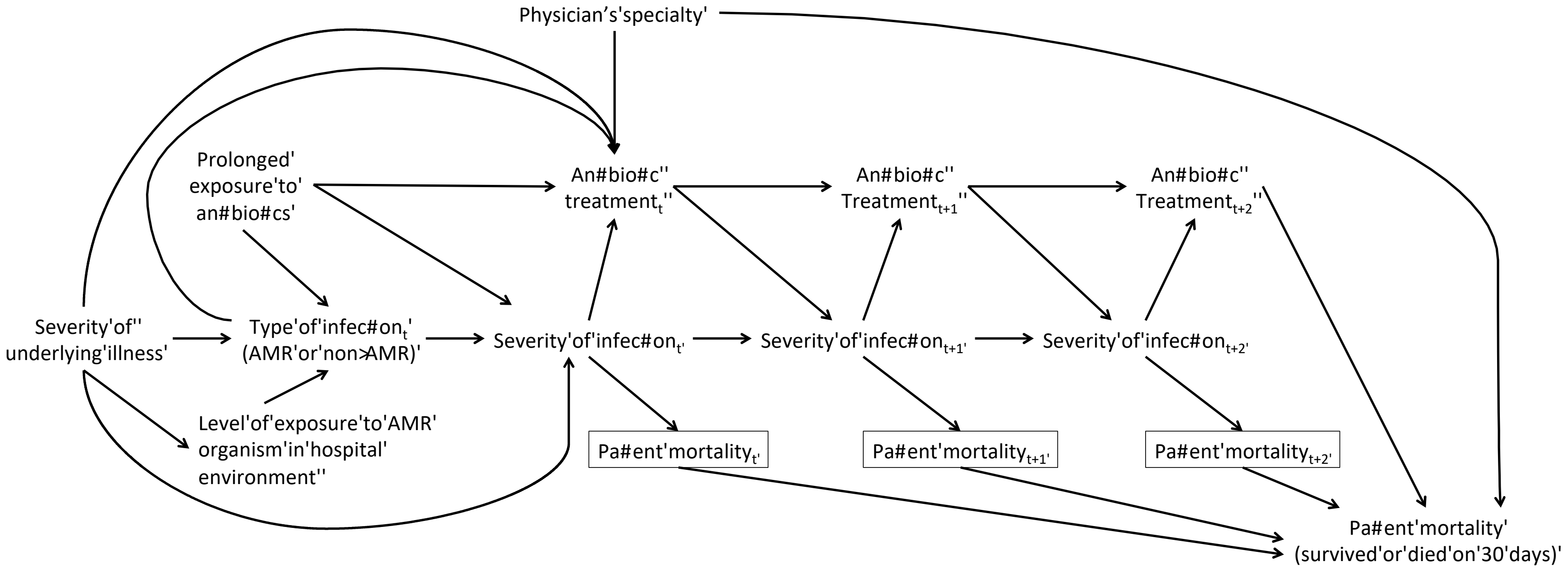

Footnote: Arrows indicate the direction of a causal relationship; for instance, the relationship "severity of underlying illnesses may cause increases in the probability of antibiotic-resistant infection" is represented by an arrow from "Severity of underlying illness" to "Type of infection (AMR or non-AMR)". The key potential baseline confounders identified were severity of underlying illness [1], antibiotic resistance pattern of the *Acinetobacter* spp. isolated from the blood sample, year in which the blood samples were collected, and specialty of the attending physician. We used the time between date of admission and date of blood sample collection, admission to intensive care unit (ICU) on the day of hospital admission, the number of days on antibiotic treatment prior to blood sample collection, and age-stratified Charlson comorbidity index (CCI) score as surrogates of severity of underlying illness. The CCI scores were calculated from the ICD 10 codes given to each patient by the attending physicians [2]. MDR *Acinetobacter* spp. was defined as previously described [3]. As data on specialty of the attending physician is not routinely collected in the electronic record, we used the department in which the patient was treated on the day of blood collection as a proxy variable. A time-varying confounder that could affect changes in empirical antibiotic treatment post blood sample collection is severity of infection, which could be affected by the history of treatment and itself may influence the decision on future treatment. The prescription of a vasopressor and transfer to ICU during the infection within the analysis time period were used to represent severity of the infection and both coded as binary time varying variables. Patient demographic information, age and gender, were also included as covariates.

Web Table 4. The estimated probability of observing detrimental outcome within 30 days post blood collection under each exposure group.

	Crude proportion of patients who developed detrimental outcome* within 30-days post blood collection	Expected proportion of patient with detrimental outcome* (95% CI) under each treatment regimen
No delays to concordant antibiotic treatment	58%	65% (57%-72%)
A one day of delays in concordant antibiotic treatment	82%	63% (50%-76%)
A two day of delays in concordant antibiotic treatment	67%	68% (56%-81%)
Three or more days of delays to concordant antibiotic treatment	50%	63% (59%-68%)

*Detrimental outcome was defined as death within the hospital, discharge without improvement, or treatment rejection within 30 days of blood collection.

Web Table 5. The estimated effect of the four treatment regimens on 30-day in-hospital all-cause mortality and detrimental outcomes.

	Odds ratio for 30-day in-hospital all-cause mortality (95% CI)	Odds ratio for detrimental outcomes* (95% CI)
No delays to concordant antibiotic treatment	Reference group	Reference group
A one day of delays in concordant antibiotic treatment	1.1 (0.6-2.1)	0.9 (0.5-1.8)
A two day of delays in concordant antibiotic treatment	1.6 (0.9-2.8)	1.2 (0.6-2.3)
Three or more days of delays to concordant antibiotic treatment	1.0 (0.7-1.5)	0.9 (0.6-1.4)

*Detrimental outcome was defined as died within the hospital, discharged without improvement, or rejected treatment within 30 days of blood collection.

Web Appendix 1. Statistical analysis and Stata code used to calculate the stabilized inverse probability weights for the marginal structural model.

Statistical analysis

We performed two analyses. The first analysis was to evaluate the impact of one or more days of delays in concordant antibiotic treatment. A propensity score for each patient was calculated to represent the probability of being prescribed with concordant antibiotic on the day of blood collection using a logistic regression model adjusting for the pre-specified confounders and using fractional polynomials to account for non-linear relationships. The propensity scores were then used to calculate stabilized inverse probability weights (IPW), which were applied to a marginal structural model. The second analysis was to evaluate the effect of one-day, two-day, and three or more days of delays in concordant antibiotic treatment. We applied two sets of IPWs to a marginal structural model. Firstly, a propensity score for each patient was calculated to represent the probability of being prescribed with a concordant antibiotic treatment on the day of blood sample collection, one day after, and two days after. The propensity scores were then used to calculate stabilized IPWs. Secondly, to emulate a randomized controlled trial with treatment regimen assigned on enrolment, patients who were discharged or died before completing the treatment were treated as censored observations, and propensity scores were calculated for being uncensored in the cohort. We then applied a marginal structural logistic regression model with the (multiplicative) total stabilized IPWs to estimate the marginal probability of 30-day mortality under each treatment regimen.

Stata code used for the analysis

```
*======================================*
* Analyses to adjust for pre-defined confounders, time-varying confounders, and immortal-time bias *
*======================================*

clear all
set more off

* ------------------- *
* Data in wide format *
* ------------------- *
use "$analysisData/hai_acine_analysis_wide.dta", clear

* Outcome variable
tab inhosp, missing
gen y=(inhosp==1 & los_spc_dis<=30)
gen sens_y=((dcstatus==3 | dctype=="2" | inhosp==1) & los_spc_dis<=30)

/*
dcstatus==3; not improved
dctype==2; rejected treatment
*/

tab y sens_y
* Exposure groups
gen exp_atb=99
replace exp_atb=0 if (strmatch(cat_name, "cat_1")==1 | ///
		strmatch(cat_name, "cat_2")==1 | strmatch(cat_name, "cat_3")==1 | ///
		strmatch(cat_name, "cat_4")==1 | strmatch(cat_name, "cat_5")==1 | ///
		strmatch(cat_name, "cat_6")==1 | strmatch(cat_name, "cat_10")==1)
replace exp_atb=1 if (strmatch(cat_name, "cat_7")==1 | ///
		strmatch(cat_name, "cat_8")==1 | strmatch(cat_name, "cat_9")==1 | ///
		strmatch(cat_name, "cat_12")==1)
replace exp_atb=2 if (strmatch(cat_name, "cat_11")==1 | ///
```

```
		strmatch(cat_name, "cat_14")==1 )
replace exp_atb=3 if (strmatch(cat_name, "cat_13")==1)
tab exp_atb, mi
tab y exp_atb, col

* IPW for t0
fp <los_adm_spc>: logit atbuse_cat0 ///
		i.MDR age i.gender_num ///
		prior_days_on_atb0 i.spc_year i.department0 ///
		i.adm_icu i.charlson_age <los_adm_spc>, or
predict ipw_deno_p1
replace ipw_deno_p1=1-ipw_deno_p1 if atbuse_cat0==0
logit atbuse_cat0
predict ipw_num_p1
replace ipw_num_p1=1-ipw_num_p1 if atbuse_cat0==0
gen ipw_p1=ipw_num_p1/ipw_deno_p1
count if ipw_p1==.
replace ipw_p1=1 if ipw_p1==.

*** Analysis: impact of ≥1 days of concordant treatment on 30-day mortality ***
preserve
* Note: if concordant then atbuse_cat0=1, otherwise 0
//gen atbuse_cat0_2=1-atbuse_cat0 if atbuse_cat0!=.
logit y i.atbuse_cat0 [pw=ipw_p1]
//margins atbuse_cat0
/*
------------------------------------------------------------------------------
             |            Delta-method
             |     Margin   Std. Err.      z    P>|z|     [95% Conf. Interval]
-------------+----------------------------------------------------------------
 atbuse_cat0 |
          0  |   .4038886    .022027    18.34   0.000     .3607165    .4470607
          1  |   .3378893   .0239133    14.13   0.000     .2910201    .3847584
------------------------------------------------------------------------------
*/
qui logit y i.atbuse_cat0 [pw=ipw_p1]
margins, dydx(atbuse_cat0)
/*
------------------------------------------------------------------------------
              |            Delta-method
              |      dy/dx   Std. Err.      z    P>|z|     [95% Conf. Interval]
--------------+----------------------------------------------------------------
1.atbuse_cat0 |  -.0659993    .032512    -2.03   0.042    -.1297217   -.0022769
------------------------------------------------------------------------------
*/
logit sens_y i.atbuse_cat0 [pw=ipw_p1]
margins atbuse_cat0
/*
------------------------------------------------------------------------------
             |            Delta-method
             |     Margin   Std. Err.      z    P>|z|     [95% Conf. Interval]
-------------+----------------------------------------------------------------
 atbuse_cat0 |
          0  |    .620404   .0222943    27.83   0.000      .576708       .6641
          1  |    .588537   .0258128    22.80   0.000     .5379448    .6391292
------------------------------------------------------------------------------
*/
logit sens_y i.atbuse_cat0 [pw=ipw_p1]
margins, dydx(atbuse_cat0)
/*
------------------------------------------------------------------------------
              |            Delta-method
              |      dy/dx   Std. Err.      z    P>|z|     [95% Conf. Interval]
--------------+----------------------------------------------------------------
1.atbuse_cat0 |   -.031867   .0341077    -0.93   0.350    -.0987169    .0349828
------------------------------------------------------------------------------
*/

restore

* IPW for t1
fp <los_adm_spc>, replace: logit atbuse_cat1 i.atbuse_cat0 i.vaso_use0##i.icu0 ///
		i.MDR age i.gender_num ///
		prior_days_on_atb0 i.spc_year i.department0 ///
		i.adm_icu i.charlson_age <los_adm_spc>, or
```

```
predict ipw_deno_p2
replace ipw_deno_p2=1-ipw_deno_p2 if atbuse_cat1==0
logit atbuse_cat1
predict ipw_num_p2
replace ipw_num_p2=1-ipw_num_p2 if atbuse_cat1==0
gen ipw_p2=ipw_num_p2/ipw_deno_p2
replace ipw_p2=1 if ipw_p2==.

* IPW for t2
fp <los_adm_spc>, replace: logit atbuse_cat2 i.atbuse_cat1 i.vaso_use1##i.icu1 ///
        i.MDR age i.gender_num ///
        prior_days_on_atb0 i.spc_year i.department0 ///
        i.adm_icu i.charlson_age <los_adm_spc>, or
predict ipw_deno_p3
replace ipw_deno_p3=1-ipw_deno_p3 if atbuse_cat2==0
logit atbuse_cat2
predict ipw_num_p3
replace ipw_num_p3=1-ipw_num_p3 if atbuse_cat2==0
gen ipw_p3=ipw_num_p3/ipw_deno_p3
replace ipw_p3=1 if ipw_p3==.

* Pooled ipw
gen sipw_tvc=(ipw_p1*ipw_p2*ipw_p3)
count if sipw_tvc==.
replace sipw_tvc=10 if sipw_tvc>10

*********************************************
*** IPW for immortal-time (Survival) bias ***
*********************************************
*** Step 1: clone ***
expand 3 if exp_atb!=0
count
bysort hn: gen n=_n
gen regimen=n
replace regimen=0 if exp_atb==0
tab regimen, mi
drop n

gen obs_full=(cat_name=="cat_1" | cat_name=="cat_4" | cat_name=="cat_5" | ///
        cat_name=="cat_7" | cat_name=="cat_10" | cat_name=="cat_11" | ///
        cat_name=="cat_12" | cat_name=="cat_13")
gen obs_mis1=(cat_name=="cat_2" | cat_name=="cat_6" | cat_name=="cat_8" | ///
                cat_name=="cat_14")
gen obs_mis2=(cat_name=="cat_3" | cat_name=="cat_9")

*** Step 2: artificial censor ***
* Censor obs that deviate from the 'assigned' regimen
gen censor=0
replace censor=1 if  (obs_full==1 & exp_atb==regimen) //1=uncensor; 0=censor
replace censor=1 if (((exp_atb==regimen) | (exp_atb+1==regimen)) & obs_mis1==1)
replace censor=1 if (obs_mis2==1) & ((exp_atb==regimen) | (exp_atb+1==regimen) |(exp_atb+2==regimen))
replace censor=1 if regimen==0

*** Step 3: IPW for censor ***
gen ipw_cen=.
* Probability of uncensored for those observed till t=2 is 1 hence IPW=1
replace ipw_cen=3 if obs_full==1 & censor==1
* Probability of uncensored for those survived till t=1
replace ipw_cen=3/2 if (cat_name=="cat_14") & (censor==1)
* Probability of uncensored for those survived >=0
replace ipw_cen=1 if (cat_name=="cat_9") & (censor==1)
* Probability of uncensored for exceptional cases
replace ipw_cen=3 if (cat_name=="cat_8") & (censor==1) & (regimen==exp_atb)
replace ipw_cen=0 if (cat_name=="cat_8") & (censor==1) & (regimen!=exp_atb)
* no weight for artifical censored obs
replace ipw_cen=0 if censor==0
* Probability of being uncensored for regimen=0
replace ipw_cen=1 if exp_atb==0 // because never artificial censored

*** Combine ipw for confounder and selection ***
gen total_ipw=ipw_cen*sipw_tvc
*** Final MSM with the total IPW ***
logit y i.regimen [pw=total_ipw], cluster(hn) or
margins regimen
/*
```

```
------------------------------------------------------------------------------
             |            Delta-method
             |     Margin   Std. Err.      z    P>|z|     [95% Conf. Interval]
-------------+----------------------------------------------------------------
     regimen |
          0  |   .3976857   .0381539    10.42   0.000     .3229055    .4724659
          1  |   .4276079   .0661868     6.46   0.000    .2978842    .5573316
          2  |   .5099309   .0617554     8.26   0.000    .3888926    .6309692
          3  |   .4089893   .0248872    16.43   0.000     .3602114    .4577673
------------------------------------------------------------------------------
*/
margins, dydx(regimen) ///difference between the margins
/*
------------------------------------------------------------------------------
             |            Delta-method
             |      dy/dx   Std. Err.      z    P>|z|     [95% Conf. Interval]
-------------+----------------------------------------------------------------
     regimen |
          1  |   .0299222   .0763964     0.39   0.695    -.1198119    .1796564
          2  |   .1122452   .0725909     1.55   0.122    -.0300304    .2545208
          3  |   .0113037   .0455531     0.25   0.804    -.0779788    .1005862
------------------------------------------------------------------------------
*/
*** Sensitivity analysis: final MSM with the total IPW ***
logit sens_y i.regimen [pw=total_ipw], cluster(hn) or
margins regimen
/*
------------------------------------------------------------------------------
             |            Delta-method
             |     Margin   Std. Err.      z    P>|z|     [95% Conf. Interval]
-------------+----------------------------------------------------------------
     regimen |
          0  |   .6463815   .0397705    16.25   0.000     .5684326    .7243303
          1  |    .632088   .0651323     9.70   0.000     .5044311     .759745
          2  |   .6842336   .0627431    10.91   0.000     .5612593    .8072078
          3  |   .6335752   .0249664    25.38   0.000      .584642    .6825084
------------------------------------------------------------------------------
*/
```

Web Figure 2. Diagnosis of the stabilized IPWs for assessing the exchangeability and positivity assumptions of the causal framework.

Distribution of stabilized IPWs used to adjust for confounders and immortal time bias in the model to estimate the marginal probability of 30-day mortality under no delays in concordant antibiotic treatment, one-day delay in concordant antibiotic treatment, two-day delay in concordant antibiotic treatment, and three or more days of delays in concordant antibiotic treatment groups.

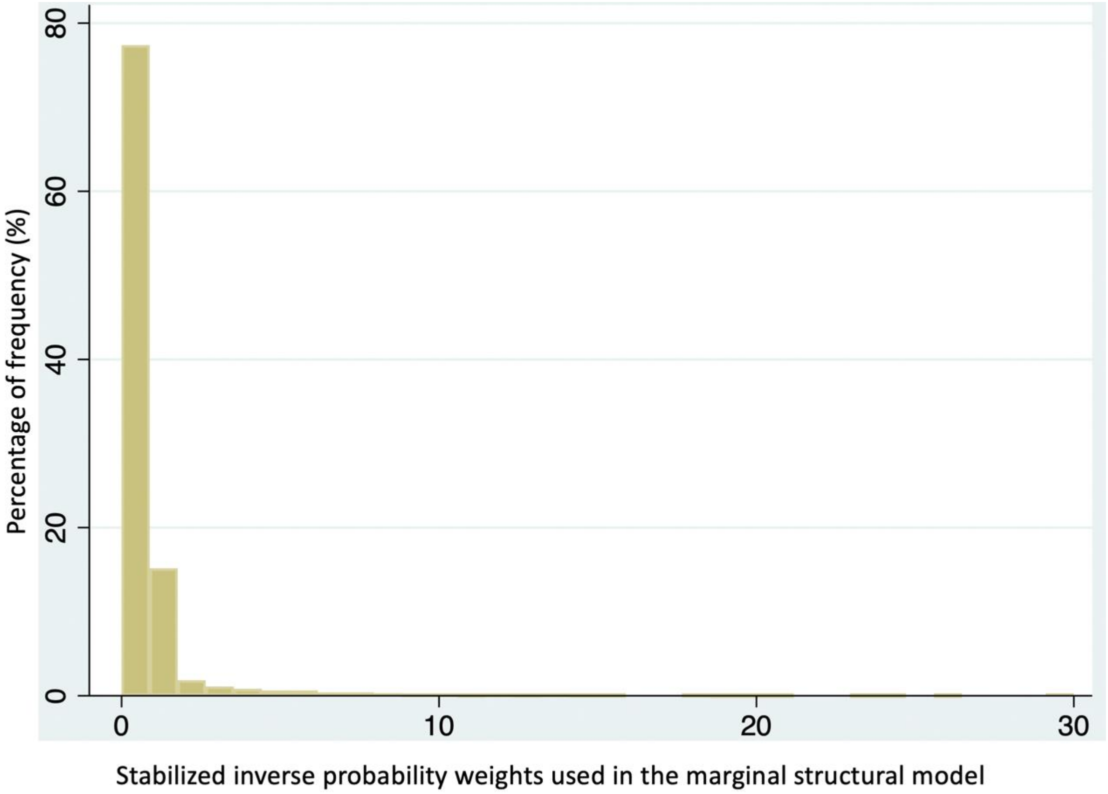

The mean and standard deviation of the stabilized IPWs were 0.889 and 2.369, respectively. The minimum and maximum stabilized IPWs were 0 and 30 respectively. The proportion of stabilized IPWs that had a value more than 5 was 0.032.

Web Table 6. Patient characteristics of the four treatment regimens in the weighted sample.

	No delays to concordant antibiotic treatment	1 day of delays in concordant antibiotic treatment	2 day of delays in concordant antibiotic treatment	At least 3 day of delays in concordant antibiotic treatment
Age (years)	55 (28-72)	57 (36-70)	48 (26-68)	54 (20-70)
Female gender	51%	49%	49%	38%
Multi-drug resistance	79%	85%	84%	74%
Age-adjusted CCI score on admission	2 (0-4)	3 (0-4)	2 (0-4)	2 (0-4)
Vasopressor prescribed on the day of blood specimen collected for culture	46%	51%	31%	32%
Admitted to ICU on the day of hospitalization	62%	66%	60%	45%
Admitted to ICU on the day of blood specimen collected for culture	65%	64%	57%	53%
Length of hospital stay since admission to blood specimen collected for culture date (days)	8 (6-16)	9 (6-17)	10 (6-18)	11 (7-18)
Days on antibiotic prior blood collection	7 (4-14)	6 (1-12)	8 (5-18)	8 (4-17)

Footnote: Age, CCI score, length of hospital stay since admission to blood collection date, and days on antibiotic prior blood specimen collection are median (IQR), and other data are n (%). ICU=intensive care unit. CCI=Charlson Comorbidity Index Score; defined using ICD10 scores [2]. Multidrug resistance is defined as non-susceptible to ≥1 agent in ≥3 antimicrobial categories [3].

Web Appendix 2. A simulation study to confirm that the three-step procedure could alleviate immortal time bias.

We consider a setting in which 2000 patients, labelled i=1, …, n, enter a study at baseline (t=0) and are randomized to treatment strategy, $regimen_{0,i}$. We assumed no confounders, and deviations from the assigned treatment were assumed to occur at random. At t=0, adherence to the assigned treatment is perfect. At t=1, patients are discharged from the hospital and the probability of being discharged depends on a factor z0, which is independent of treatment assigned, and also depends on the survival status of the patient. At t=2, z1 determines the probability of being discharged, which also depends on the survival status of the patient. The simulations showed biases in the estimation of the expected mortality using a standard logistic regression model. Such bias is alleviated using the three-step procedure described by Hernan 2018 (9). The simulation was performed in STATA 15.1 (StataCorp LP, College station, Texas, USA). The code used is presented below.

```
*********************
*** Simulated dataset ***
*********************
clear all

set seed 2009
set obs 2000

*** Assign unique identifier
egen hn=seq(), f(1) t(2000)
*** True regimen (that would have randomised and recevied in a RCT)
egen regimen=seq(), f(1) t(4)
tab regimen
*** Dummy variables for the regimens
gen dummy0=(regimen==1)
gen dummy1=(regimen==2)
gen dummy2=(regimen==3)
gen dummy3=(regimen==4)
* Factor that influence the discharge rate
gen z0=uniform()<0.6
tab z0
*** True survival status
gen y=uniform()<0.3+0.05*dummy1+0.1*dummy2+0.15*dummy3+0.5*z0
tab y regimen, col
qui logit y i.regimen i.z0, or
margins regimen
/*
------------------------------------------------------------------------------
             |            Delta-method
             |     Margin   Std. Err.      z    P>|z|     [95% Conf. Interval]
-------------+----------------------------------------------------------------
     regimen |
          1  |   .5963613   .0188126    31.70   0.000     .5594894    .6332333
          2  |   .6616943   .0184831    35.80   0.000     .6254682    .6979205
          3  |   .7084723   .0175678    40.33   0.000     .6740401    .7429045
          4  |   .7314503    .017665    41.41   0.000     .6968276     .766073
------------------------------------------------------------------------------
*/

*** Probability of getting discharged
*** Daily antibiotic treatment, where 1=concordant; 0=discordant; NA=discharged
* Day 0
gen atbuse_cat0=(regimen==1)
tab regimen atbuse_cat0
* Day 1: the probability of remaining in the hospital depends on y and z0
gen atbuse_cat1=1 if ///
        uniform()<[dummy0*(1) + dummy1*(0.9-0.5*y-0.5*z0) + dummy2*(-1) + dummy3*(-1)]
replace atbuse_cat1=0 if ///
        uniform()<[dummy0*(-1) + dummy1*(-1) + dummy2*(0.9-0.5*y-0.5*z0) + dummy3*(0.9-0.5*y-0.5*z0)]
tab atbuse_cat1 regimen, col mis

* Factor that influence the discharge rate
gen z1=uniform()<0.5
tab z1
* Day 2
gen atbuse_cat2=1 if ///
        atbuse_cat1!=. & uniform()<[dummy0*(1) + dummy1*(1) + dummy2*(0.9-0.5*y-0.1*z1) + dummy3*(-1)]
replace atbuse_cat2=0 if ///
```

```
        atbuse_cat1!=. & uniform()<[dummy0*(-1) + dummy1*(-1) + dummy2*(-1) + dummy3*(0.9-0.5*y-0.1*z1)]
tab atbuse_cat2 regimen, col mis
drop dummy*

*** Re-label the individuals
gen cat_name="cat_1" if (atbuse_cat0+atbuse_cat1+atbuse_cat2)==3
replace cat_name="cat_2" if  (atbuse_cat0==1 & atbuse_cat1==1 & atbuse_cat2==.)
replace cat_name="cat_3" if  (atbuse_cat0==1 & atbuse_cat1==. & atbuse_cat2==.)
replace cat_name="cat_4" if  (atbuse_cat0==1 & atbuse_cat1==1 & atbuse_cat2==0)
replace cat_name="cat_5" if  (atbuse_cat0==1 & atbuse_cat1==0 & atbuse_cat2==1)
replace cat_name="cat_6" if  (atbuse_cat0==1 & atbuse_cat1==0 & atbuse_cat2==.)
replace cat_name="cat_7" if  (atbuse_cat0==0 & atbuse_cat1==1 & atbuse_cat2==1)
replace cat_name="cat_8" if  (atbuse_cat0==0 & atbuse_cat1==1 & atbuse_cat2==.)
replace cat_name="cat_9" if  (atbuse_cat0==0 & atbuse_cat1==. & atbuse_cat2==.)
replace cat_name="cat_10" if  (atbuse_cat0==1 & atbuse_cat1==0 & atbuse_cat2==0)
replace cat_name="cat_11" if  (atbuse_cat0==0 & atbuse_cat1==0 & atbuse_cat2==1)
replace cat_name="cat_12" if  (atbuse_cat0==0 & atbuse_cat1==1 & atbuse_cat2==0)
replace cat_name="cat_13" if  (atbuse_cat0==0 & atbuse_cat1==0 & atbuse_cat2==0)
replace cat_name="cat_14" if  (atbuse_cat0==0 & atbuse_cat1==0 & atbuse_cat2==.)
tab cat_name y, ro

*** Re-define the exposure
gen exp_atb=99
replace exp_atb=1 if (strmatch(cat_name, "cat_1")==1 | ///
        strmatch(cat_name, "cat_2")==1 | strmatch(cat_name, "cat_3")==1 | ///
        strmatch(cat_name, "cat_4")==1 | strmatch(cat_name, "cat_5")==1 | ///
        strmatch(cat_name, "cat_6")==1 | strmatch(cat_name, "cat_10")==1)
replace exp_atb=2 if (strmatch(cat_name, "cat_7")==1 | ///
        strmatch(cat_name, "cat_8")==1 | strmatch(cat_name, "cat_9")==1 | ///
        strmatch(cat_name, "cat_12")==1)
replace exp_atb=3 if (strmatch(cat_name, "cat_11")==1 | ///
        strmatch(cat_name, "cat_14")==1 )
replace exp_atb=4 if (strmatch(cat_name, "cat_13")==1)
tab regimen exp_atb, mi
tab y exp_atb, col

logit y i.exp_atb, or
margins exp_atb
/*
------------------------------------------------------------------------------
             |            Delta-method
             |     Margin   Std. Err.      z    P>|z|     [95% Conf. Interval]
-------------+----------------------------------------------------------------
     exp_atb |
          1  |       .592   .0219789    26.93   0.000     .5489221    .6350779
          2  |    .812605   .0113122    71.83   0.000     .7904336    .8347765
          3  |   .3317757   .0321867    10.31   0.000     .2686909    .3948605
          4  |     .15625   .0370579     4.22   0.000     .0836178    .2288822
------------------------------------------------------------------------------
*/

*** Recode the regimen and exp_atb variables
gen true_exp=regimen-1
gen obs_exp=exp_atb-1
drop regimen
drop exp_atb
rename obs_exp exp_atb

* Analysis based on regimen completed population
preserve
drop if atbuse_cat1==. | atbuse_cat2==.
count
logit y i.exp_atb, or
margins exp_atb
restore

* IPW for immortal bias
*** Step 1: clone ***
expand 3 if exp_atb!=0
count
bysort hn: gen n=_n
gen regime=n
replace regime=0 if exp_atb==0
tab regime, mi
drop n
```

```
gen obs_full=(cat_name=="cat_1" | cat_name=="cat_4" | cat_name=="cat_5" | ///
              cat_name=="cat_7" | cat_name=="cat_10" | cat_name=="cat_11" | ///
              cat_name=="cat_12" | cat_name=="cat_13")
gen obs_mis1=(cat_name=="cat_2" | cat_name=="cat_6" | cat_name=="cat_8" | ///
                             cat_name=="cat_14")
gen obs_mis2=(cat_name=="cat_3" | cat_name=="cat_9")

*** Step 2: artifical censor ***
* Censor obs that deviate from the 'assigned' regime
gen censor=0
replace censor=1 if  (obs_full==1 & exp_atb==regime) //1=uncensor; 0=censor
replace censor=1 if (((exp_atb==regime) | (exp_atb+1==regime)) & obs_mis1==1)
replace censor=1 if (obs_mis2==1) & ((exp_atb==regime) | (exp_atb+1==regime) |(exp_atb+2==regime))
replace censor=1 if regime==0

*** Step 3: IPW for censor ***
gen ipw_cen=.
* Probability of uncensored for those observed till t=2 is 1 hence IPW=1
replace ipw_cen=3 if obs_full==1 & censor==1
* Probability of uncensored for those survived till t=1
replace ipw_cen=3/2 if (cat_name=="cat_14") & (censor==1)
* Probability of uncensored for those survived >=0
replace ipw_cen=1 if (cat_name=="cat_9") & (censor==1)
* Probability of uncensored for exceptional cases
replace ipw_cen=3 if (cat_name=="cat_8") & (censor==1) & (regime==exp_atb)
replace ipw_cen=0 if (cat_name=="cat_8") & (censor==1) & (regime!=exp_atb)
replace ipw_cen=0 if censor==0
* Probability of being uncensored for regime=0
replace ipw_cen=1 if exp_atb==0 // because never artificial censored

logit y i.regime [pw=ipw_cen], cluster(hn) or
margin regime
/*
------------------------------------------------------------------------------
             |            Delta-method
             |     Margin   Std. Err.      z    P>|z|     [95% Conf. Interval]
-------------+----------------------------------------------------------------
      regime |
          0  |       .592   .0219844    26.93   0.000     .5489114    .6350886
          1  |    .690604   .0174631    39.55   0.000     .6563769    .7248311
          2  |   .6911813   .0166179    41.59   0.000     .6586107    .7237518
          3  |   .7248049   .0162734    44.54   0.000     .6929095    .7567003
------------------------------------------------------------------------------
*/
```

Web Table 7. Assessing our current study and the four articles identified with a similar study objective using the Risk of Bias in Non-randomised Studies of Interventions (ROBINS-I) [4].

**Study***	**Bias due to confounding**	**Bias in selection of participants into the study**	**Bias in classification of interventions**	**Bias due to deviations from intended interventions**	**Bias due to missing data**	**Bias in measurement of outcomes**	**Bias in selection of reported result**	**Overall risk of bias**
Our current study	Low risk of bias	Low risk of bias	Low risk of bias	Low risk of bias	Low risk of bias	Low risk of bias	Low risk of bias	Low risk of bias
Zilberberg MD, et al. 2016 [5]	Serious risk of bias	Moderate risk of bias	Serious risk of bias	Low risk of bias	No information	Low risk of bias	Low risk of bias	Serious risk of bias
Al-Dorzi HM, et al. 2015 [6]	No information	No information	Serious risk of bias	Low risk of bias	Low risk of bias	Low risk of bias	Serious risk of bias	Serious risk of bias
Shorr AF, et al. 2014 [7]	Serious risk of bias	No information	Low risk of bias	Low risk of bias	Low risk of bias	Low risk of bias	Low risk of bias	Serious risk of bias
Kuo SC, et al. 2013 [8]	Serious risk of bias	Moderate risk of bias	Serious risk of bias	Low risk of bias	No information	Low risk of bias	Serious risk of bias	Serious risk of bias

Footnote: To review the current evidence for the impact of delays in concordant antibiotic treatment on mortality, we searched PubMed using the search term: (((((Acinetobacter [TITLE]) AND ((("antibiotic" or "antibiotics" or "antimicrobial therapy" or "empiric therapy") and ("mortality" or "death" or "failure" or "survival") and ("bloodstream" or "bacteremia" or "bacteraemia" or "septicemia" or "sepsis") and ("inappropriate" or "appropriate" or "discordant" or "non concordant" or "concordant" or "delayed" or "covering" or "noncovering")))))))), with no language restriction. We found 77 articles that were published before December 2019. We found no studies that aimed to evaluate the impact of delays in concordant empirical antibiotic treatment among patients with *Acinetobacter* spp. bacteremia in low and middle-income countries (LMICs). We found four studies that aimed specifically to evaluate the relationship between inappropriate empirical antibiotics and mortality in high-income countries [5-8]. Three of these four studies reported that appropriate empirical antibiotic therapy was associated with a reduction of in relative risk of mortality, with reductions from 42-85% [5-7]. One study reported no association [8]. The proportion of isolates that were carbapenem resistant rate was reported as 18% is one study [7], 58% in another [6], and not reported in the other two [5, 6]. The largest study was performed in the United States on a cohort of 1423 patients with community-acquired *Acinetobacter baumannii* pneumonia and sepsis [4]. The remaining three studies had sample sizes of fewer than 270 patients. Of the remaining 73 studies, 39 articles written in English were relevant and sought to identify “risk factors” for mortality among patients with *Acinetobacter* spp. m, but did not explicitly set out to answer causal questions. These studies tended to be small (sample sizes ranged from 26 to 399, IQR 54-177) and, while many reported associations of mortality with inappropriate empirical antibiotic treatment, these studies used methods that would not be expected to yield causally interpretable results.

References

1. Limmathurotsakul D, Dunachie S, Fukuda K, et al. Improving the estimation of the global burden of antimicrobial resistant infections. Lancet Infect Dis 2019;19:e392-8.

2. Sundararajan V, Henderson T, Perry C, Muggivan A, Quan H, Ghali WA. New ICD-10 version of the Charlson comorbidity index predicted in-hospital mortality. J Clin Epidemiol 2004;57:1288-94.

3. Magiorakos AP, Srinivasan A, Carey RB, et al. Multidrug-resistant, extensively drug-resistant and pandrug-resistant bacteria: an international expert proposal for interim standard definitions for acquired resistance. Clin Microbiol Infect 2012;18:268-81.

4. Sterne JA, Hernan MA, Reeves BC, et al. ROBINS-I: a tool for assessing risk of bias in non-randomised studies of interventions. BMJ 2016;355:i4919.

5. Zilberberg MD, Nathanson BH, Sulham K, Fan W, Shorr AF. Multidrug resistance, inappropriate empiric therapy, and hospital mortality in Acinetobacter baumannii pneumonia and sepsis. Crit Care 2016;20(1):221.

6. Al-Dorzi HM, Asiri AM, Shimemri A, et al. Impact of empirical antimicrobial therapy on the outcome of critically ill patients with Acinetobacter bacteremia. Ann Thorac Med 2015;10(4):256-62.

7. Shorr AF, Zilberberg MD, Micek ST, Kollef MH. Predictors of hospital mortality among septic ICU patients with Acinetobacter spp. bacteremia: a cohort study. BMC infect Dis 2014;14:572.

8. Kuo SC, Lee YT, Yang SP, et al. Evaluation of the effect of appropriate antimicrobial therapy on mortality associated with Acinetobacter nosocomialis mia. Clin Microbiol Infect 2013;19(7):634-9.
